# Supplementary material for: Content-rich biological network constructed by mining PubMed abstracts
Source: BMC Bioinformatics. 2004 Oct 8;5:147. doi: 10.1186/1471-2105-5-147 (PMC528731; doi:10.1186/1471-2105-5-147)
Supplement: Additional File 2 — The original results of the above study (non-essential files are deleted to keep the file size under the limit set by BMC bioinformatics). [file 1471-2105-5-147-S2.bz2 › chilibotAdditionalFile2/dip05/232ID7931156E751/html/UL9_UL8.html]

 


 **UL9** and **UL8** 
  
Found 20 abstracts in PubMed, retrieved 05.  
 

 What does Google say? 
 PDF only 
| .edu only 

---

**Interactive relationship** (e.g. stimulation, inhibition, etc)

**Neutral relationship**- Analysis of transcripts and time course studies demonstrated that the  **UL8**  protein is translated from a delayed early expressed 3.1 kb polyadenylated mRNA which initiates within the  **UL9**  ORF.  Ref: 9460934 J Gen Virol, 1998
- A truncated protein, consisting of the C terminal 487 amino acids of OBP  [ **UL9** ] , termed OBPC, is the product of the HSV  **UL8** .5 gene and binds to origin sequences, although OBPC s role in HSV DNA replication is not yet clear.  Ref: 11435559 J Virol, 2001

**Non-interactive relationship** (e.g. studied together, co-existance, homology, etc.)

- Since this pattern can form in cells infected with mutants which are defective in UL5,  **UL8** ,  **UL9** , or UL52 in the presence of polymerase inhibitors C.  Ref: 9151871 J Virol, 1997
- The results shown in this paper are consistent with the model that the five proteins, ICP8, UL5,  **UL8** ,  **UL9** , and UL52, form a scaffold.  Ref: 12634381 J Virol, 2003
- The ordered assembly of the herpes simplex virus HSV type 1 replication apparatus leading to replication compartments likely involves the initial assembly of five viral replication proteins, ICP8,  **UL9** , and the heterotrimeric helicase primase complex UL5  **UL8**  UL52, into replication foci.  Ref: 12634381 J Virol, 2003
